# Production of Antioxidant and ACEI Peptides from Cheese Whey Discarded from Mexican White Cheese Production

**DOI:** 10.3390/antiox8060158

**Published:** 2019-06-03

**Authors:** Sandra Teresita Martín-del-Campo, Pablo César Martínez-Basilio, Juan Carlos Sepúlveda-Álvarez, Susana Estela Gutiérrez-Melchor, Karla Deniss Galindo-Peña, Ana Karen Lara-Domínguez, Anaberta Cardador-Martínez

**Affiliations:** Tecnologico de Monterrey, Escuela de Ingeniería y Ciencias, Centro de Bioingeniería, Epigmenio González 500, Fracc. San Pablo, Querétaro Qro. 76130, Mexico; pablo1903.mb@gmail.com (P.C.M.-B.); A01065086@itesm.mx (J.C.S.-Á.); A01420054@itesm.mx (S.E.G.-M.); A00988581@itesm.mx (K.D.G.-P.); A00367451@itesm.mx (A.K.L.-D.)

**Keywords:** cheese whey, trypsin hydrolysis, antioxidant capacity, ACEI activity

## Abstract

Cheese whey, a byproduct of the cheese-making industry, is discarded in many countries in the environment, causing pollution. This byproduct contains high-quality proteins containing encrypted biologically active peptides. The objective of this work was to evaluate the suitability of using this waste to produce bioactive peptides by enzymatic hydrolysis with a digestive enzyme. Cheese whey from white cheese (Panela cheese) was concentrated to increase total protein and hydrolyzed with trypsin. A central composite design was used to find the best conditions of pH and temperature, giving the higher antioxidant capacity and Δ Angiotensin-converting enzyme inhibition (Δ ACEI) activity. Higher biological activities were found when hydrolysis was performed at 52 °C and a pH of 8.2. The maximum value for the 2,2- diphenyl-1-picrylhydrazyl (DPPH)-scavenging activity was 26%, while the higher Δ ACE inhibition was 0.89. Significant correlations were found between these biological activities and the peptides separated by HPLC. The hydrophilic fraction (HI) showed highly significant correlations with the antioxidant capacity (*r* = 0.770) and with Δ ACE inhibition (*r* = 0.706). Antioxidant capacity showed a significant positive correlation with 34 peaks and Δ ACE inhibition with 33 peaks. The cheese whey was successfully used as raw material to produce peptides showing antioxidant capacity and ACEI activity.

## 1. Introduction

Currently, diverse food industries produce waste products with high nutritional value, mostly in the form of proteins and minerals. Cheese whey is a product with high nutritional value since it contains lactose, minerals, vitamins, and proteins of high nutritional value [1]. Several components of cheese whey could be used for the production of products of nutritional, medicinal, or industrial interest. The main whey proteins are β-lactoglobulin and α-lactalbumin [2]. Most of the waste products are not used and are discarded to the environment, causing pollution. Whey is the main byproduct of the cheese-making industry, accounting for 85–90% of the milk volume [1] used as raw material. According to the FAO [3], in 2014, the world produced 22,651,605 tons of cheese, representing production of more than 220 million tons of whey, but only 3,151,924 tons of whey products are produced. Despite its nutritional value, about 47% of cheese whey is discharged to the drain and reaches rivers and soils, causing serious pollution problems [1]. In Mexico, panela (white) cheese production represents 15% of total cheese production [4].

At present, proteins and peptides with biological activity constitute one of the most important categories within the functional food sector. Peptides with biological activity can be generated from milk proteins [5,6]. Casein encrypted peptides show higher biological activities than whey encrypted peptides [7,8]. Nevertheless, the whey encrypted peptides released by enzymatic [2,9,10] or microbiologic hydrolysis [11] present different biological activities. Among the biological activities of those peptides, the antioxidant capacity [12] can reduce oxidative stress. β-lactoglobulin peptides can pass through the intestinal barrier to reach more distant cells [13]. Moreover, the antioxidant activity of whey protein hydrolysates depends on peptide molecular weight, with low-molecular-weight peptides (0.1–2.8 kD) showing the strongest in vitro radical scavenging activity [14]. Angiotensin-converting enzyme inhibition (ACEI) [2,7,9,11,12,15] has been shown by β-lactoglobulin, which lowers blood pressure [16]. The antibacterial activity of whey peptides has been studied by several authors [2,12,17], for instance, Osman et al. [18] demonstrated that goat whey peptides had bactericidal activity against both Gram-positive and Gram-negative bacteria. According to Brandelli, Daroit, and Corrêa [5] and Teixeira et al. [19], whey diet supplementation represents a practical, feasible, and cost-effective approach to mitigate cancer cachexia syndrome, due to its potential to inhibit incidence and growth of cancer cells. For instance, whey peptides could decrease the proliferation of colon cancer cells as reported by De Simone et al. [20]. Cholesterol-lowering effects have also been noted as a result of changes in micellar cholesterol solubility in the intestine [2]. It is reported that oral administration of whey peptides positively affects not only blood glucose control, but also insulinotropic responses in human and animal models [5]. Furthermore, the α-lactalbumin of camel whey can generate potent peptides that inhibit dipeptidyl peptidase IV (DPP-IV), which could increase insulin secretion during the postprandial phase, allowing for a better glycemic regulation [21,22]. 

To produce bioactive peptides, most research has been carried out with digestive enzymes at their optimal conditions [2,7,10] using commercial dry cheese whey [10], purified whey proteins [2,23,24], or microfiltration permeates [7]. 

To our knowledge, there are no studies about the production of bioactive peptides from the cheese whey discarded during the production of panela cheese. This work aimed to evaluate the suitability of using the whey resulting from Mexican white cheese to produce antioxidant and antihypertensive peptides by enzymatic hydrolysis.

## 2. Materials and Methods 

### 2.1. Cheese Whey Production and Conditioning

Sweet cheese whey for this study was obtained from panela (white) cheese production. Cheese production was performed at pilot scale as described by Guerra Martínez et al. [25] in the Tecnologico de Monterrey facilities (Querétaro, Mexico) under controlled conditions with milk obtained in the Tecnológico de Monterrey experimental agricultural field (CAETEC) (Querétaro, Mexico). One 20 L batch of cheese whey was collected just after the first curd draining and was frozen at −18 °C in tightly closed containers to stop the biochemical and microbiological reactions until conditioning.

Conditioning consisted of whey concentration and skimming. Concentration was performed in a rotary evaporator under vacuum at 60 °C to reach an approximate final protein concentration of 7 mg/mL. Protein content was evaluated with the Bradford method described in Section 2.4. Next, concentrated cheese whey was skimmed after 1 h cooling at 5 °C by manual separation of the fat layer. Then, samples were frozen at −20 °C until hydrolysis. 

### 2.2. Enzymatic Hydrolysis Experimental Design

Cheese whey hydrolysis was performed with Trypsin (1:250) from bovine pancreas (Difco Laboratories, Detroit, Mich., USA) solution at 0.65 g/L in Tris-HCl 0.02 N (Sigma-Aldrich, St. Louis, MO, USA) CaCl_2_ 0.01 M (Meyer, Química Suastes, Ciudad de México, Mexico) pH 8 buffer. 

A Central composite rotatable design was selected for the enzymatic hydrolysis experiment. Evaluated factors were pH (7.5, 9.0) and temperature (37 °C, 50 °C), default central points, and two replicates were performed. Evaluated response variables were hydrolysis degree, soluble protein, antioxidant capacity, angiotensin-converting enzyme inhibition, as well as peptides profiles, amino acid, hydrophobic, and hydrophilic peptides fractions. After thawing at 5 °C and homogenization, the protein content in the cheese whey was measured to dose the enzyme at a ratio of 5:100 enzyme–substrate. pH was adjusted before enzyme addition, and the temperature was controlled by incubation in a MaxQ Shaker (Thermo Scientific, Marietta, OH, USA). Hydrolysis was carried out for 1 h and then stopped by incubating at 95 °C for 15 min. Samples were analyzed to evaluate hydrolysis degree and soluble protein, then they were frozen at –18 °C until further analysis.

### 2.3. Degree of Hydrolysis

The degree of hydrolysis was measured according to Nielsen et al. [26] using o-phthaldialdehyde (OPA) reagent. The OPA reagent was prepared as follows: 7.620 g di-Na-tetraborate decahydrate and 200 mg Na-dodecyl-sulfate were dissolved in 150 mL deionized water. Once those reagents were completely dissolved, 160 mg 97% (OPA, Sigma-Aldrich) dissolved in 4 mL ethanol was transferred quantitatively to the previous solution. 176 mg dithiothreitol 99% was added to the solution. Finally, the solution was made up to 200 mL with deionized water. The reaction was prepared as follows: 3 mL of OPA was mixed with 400 µL of sample, let stand exactly two minutes at room temperature (25 °C) and absorbance was read at 340 nm with a spectrophotometer (Labomed, Inc. Spectro UV-VIS Double Beam UVD-3500, Culver, Ca, U.S.A.). Serine dissolved in MilliQ water (0.9516 meqv/L) was used as a standard. Blanks were prepared using MilliQ water instead of a sample. Degree of hydrolysis was calculated according to the equation given by Nielsen, Petersen, and Dambmann [26].

Serine-NH_2_ = (ODsample − Odblank)/(ODstandard − Odblank) × 0.9516 meqv/L × 0.1 × 100/ X × P (1)

where serine-NH_2_ = meqv serine NH_2_/g protein; X = g sample; P = protein% in sample; 0.1 is the sample volume in liter (L).

h = (serine-NH_2_-β)/α meqv/g protein (2)

DH = h/htot × 100%(3)

Values for α, β, and htot were 1, 0.4, and 8.5, respectively according to the values for whey given by Nielsen, Petersen and Dambmann [26].

### 2.4. Total and Soluble Protein

Before evaluation of soluble protein, hydrolyzed samples were centrifuged at 4 °C and 5000 rpm for 10 min to eliminate insoluble aggregates. Protein was evaluated with the Bio-Rad microplate protocol based on the Bradford method. Absorbance at 595 nm was obtained in a Microplate Spectrophotometer (Bio-Rad xMark Microplate Spectrophotometer, Tokio, Japan). Bovine serum albumin (Sigma-Aldrich) was used as a calibration standard. 

### 2.5. Antioxidant Capacity by DPPH Method

The method of Oomah et al. [27] was used to assess the 2,2- diphenyl-1-picrylhydrazyl (DPPH, Sigma-Aldrich) radical-scavenging activity of hydrolyzed whey protein. An aliquot of 20 µL of whey was mixed with 220 µL of 80% methanolic solution of DPPH (125 µM). The absorbance of the resulting solutions was measured at 517 nm after 90 min using a visible-UV microplate reader (680 XR Microplate Reader, Bio-Rad Laboratories, Inc., Tokio, Japan). The results were expressed as percent DPPH discoloration [28]. Samples were analyzed in triplicate.

### 2.6. Reverse-Phase HPLC of Hydrolyzed Whey Protein

Hydrolyzed whey was filtered through a 0.45 µm filter (PTFE Syringe Filter, Agilent, Palo Alto, CA, USA). Reverse Phase High Performance Liquid Chromatography (RP-HPLC) was performed using a high-performance liquid chromatography Agilent 1200 system (Agilent). The HPLC system was comprised of a solvent degasser, a quaternary pump, an autosampler, and a wavelength ultraviolet-visible detector. A RP-C18 column (Zorbax XDB 5 µm × 4.6 × 150 mm, Agilent) was selected and kept at room temperature (25 °C). Sample volume was 20 µL, and the flow rate was 0.5 mL/min. Eluent A was 0.05% TFA. Eluent B was 60% acetonitrile containing 0.05% TFA and 49.95% water. A linear gradient was applied from 0 to 80% eluent B over 100 min. The UV-visible detector was set at 215 nm. Samples were analyzed in triplicate.

The area of each peak in the chromatogram was used as a measure of the peak concentration. In the obtained chromatograms, the resulting peaks were divided according to the retention time into three fractions to generate three data sets. The first data set consisted of the compounds eluting during the first 10 min, where most of the free amino acids eluted (AA fraction). For the other two data sets, the criteria of Gonzalez De Llano et al. [29] was followed. The hydrophilic (HI) fraction contained peaks between 10 and 35 min, while the hydrophobic (HO) fraction contained those from 35 to 120 min. Additionally, the ratio between HI:HO was obtained using the total area of each fraction in each chromatogram.

### 2.7. Angiotensin-Converting Enzyme Inhibition Assay (ACE)

ACE inhibition activity was determined with the method of Wang et al. [30] using reverse-phase high-performance liquid chromatography (RP-HPLC). This method is based on the hippuric acid (HA) liberation from hippuryl-histidyl-leucine (HHL) catalyzed by ACE. ACE 0.1 U/mL (from porcine kidney, 0.5 U, Sigma-Aldrich) and HHL 3 mM (Sigma-Aldrich) were prepared by dissolution in 100 mM borate buffer (pH 8.3) with 300 mM NaCl. After a preincubation at 30 °C for 30 min, an aliquot of 25 µL hydrolyzed whey was added with 25 µL of ACE and kept at 37 °C for 10 min; next, 25 µL of HHL was added. After incubation, to stop the reaction, 83.5 µL of HCl 0.1 M was added. 

Samples were analyzed by RP-HPLC in an Agilent Technologies 1200 series (Palo Alto, CA, USA) with a diode array detector (DAD) set at 226 nm, and a Zorbax Eclipse XDB-C18 column (5 μm, 4.6 μm i.d. × 150 mm, Agilent, USA) at 25 °C. Sample volume was 5 µL, and the flow rate was 0.5 mL/min. Solvents were as follows: (A) 0.5 mL/L TFA in HPLC-grade water. (B) Acetonitrile. The ratio of solvents A/B was 7:3 in an isocratic flow. HA was quantified using a calibration curve (0–1 mM).

The peptide’s ACE inhibition activity was compared against Captopril (CAS 62571-86-2). Captopril (Sigma-Aldrich) solution of 5 mg/mL (23.01 mM) was analyzed under the same conditions. ACE inhibition activity of hydrolyzed samples is reported as the ratio of the peptides ACE inhibition activity against Captopril activity (Δ ACE inhibition). Samples were analyzed in triplicate.

### 2.8. Statistical Analysis

Statistical analysis was performed with STATISTICA v13 (TIBCO Software Inc, Palo Alto, CA, USA). Analysis of Variance (ANOVA) was applied to identify significant effect (*p* < 0.05) of pH or temperature over the % Hydrolysis, Soluble Protein, Antioxidant capacity, Δ ACE inhibition, as well as AA, Hydrophilic (HI) and Hydrophobic fractions (HO), and HI:HO ratio. Next, simple linear correlation analysis (Pearson’s *r*) was applied to identify significant correlations (*p* < 0.05) among biological activities with peptide fractions and/or individual peaks in the chromatograms.

## 3. Results

### 3.1. Analysis of Variance (ANOVA)

ANOVA showed that pH and temperature had significant effects on different parameters (Table 1). pH showed significant effects (*p* < 0.05) on the hydrolysis (% hydrolysis and soluble protein) and the evaluated biological activities (antioxidant capacity and Δ ACE inhibition activity) but not on the hydrolysis products. On the other hand, the temperature only showed significant effects on soluble protein, antioxidant capacity, and the hydrophilic fraction.

**Figure 1:**
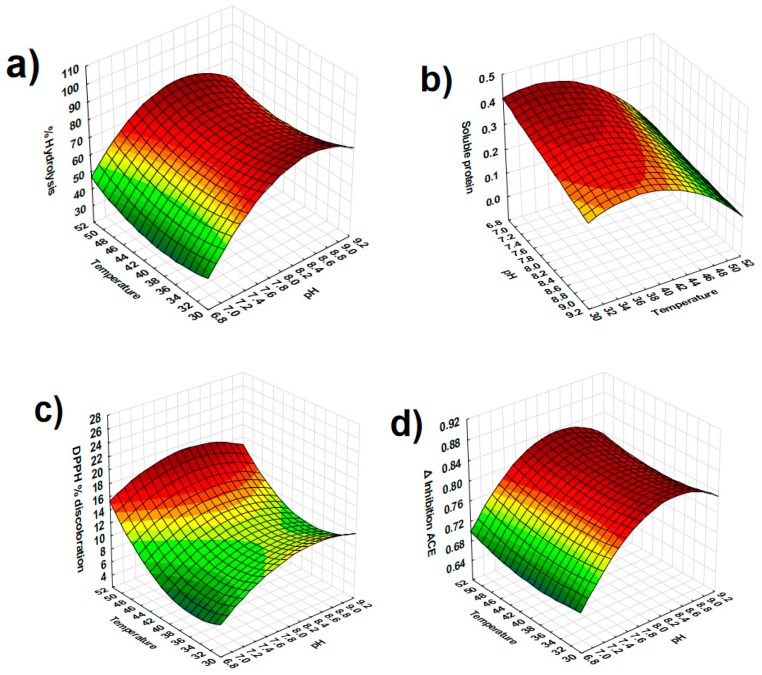

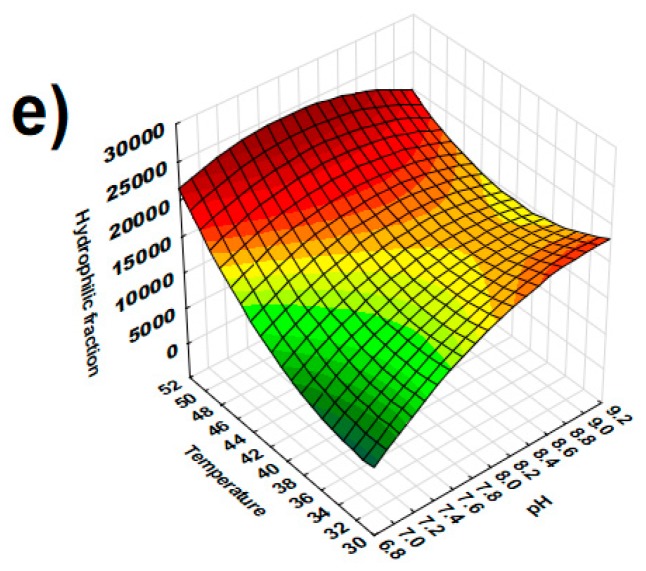
Response surface plots of pH and temperature for (**a**) % of hydrolysis, (**b**) soluble protein (mg/mL), (**c**) antioxidant capacity (DPPH % discoloration), (**d**) Δ ACE inhibition, and (**e**) peptides hydrophilic fraction (HI).

Figure 1 shows the surface plots for the response parameters with at least one significant factor. % Hydrolysis increased as pH increased (Figure 1a) while soluble protein presented higher values at low pH and low temperature (Figure 1b). 

Both antioxidant capacity and ACE inhibition showed higher activity at 52 °C and pH 8.2 (Figure 1c,d). These conditions of pH and temperature also produced a significantly higher concentration of HI peptides (Figure 1e). 

### 3.2. Correlation Analysis

Simple linear correlation analysis made it possible to identify significant (*p* < 0.05) correlations between the hydrolyzed samples’ antioxidant capacity and ACEI activity with the individual peptides and peptide fractions obtained by HPLC. A total of 128 peaks were registered and coded as Px, where x is a consecutive number according to the retention time. Results of significant correlations with at least one of the biological activities are shown in Table 2. 

Antioxidant capacity and ACEI inhibition showed significant and positive correlation between them and a highly significant positive correlation (*p* < 0.01) with the hydrophilic fraction (HI). Only 18 peaks showed significant correlations with both biological activities at the same time.

Antioxidant capacity showed a significant positive correlation with 25 peaks of the hydrophilic and AA fractions and a highly significant (*p* < 0.01) negative correlation with the P3 and P75. For the hydrophobic fraction, a significant positive correlation was observed for nine peaks.

On the other hand, ACEI activity showed a significant positive correlation with 25 peaks of the hydrophilic and AA fractions and a significant (*p* < 0.05) negative correlation with P62; additionally, significant positive correlations was found for eight peaks in the hydrophobic fraction. 

## 4. Discussion

The highest content of soluble protein was obtained at the lowest temperature, but the % hydrolysis was not affected by this parameter. This could be due to the proteins unfolding and aggregates forming due to the temperature, which on one hand reduces protein solubility, but on the other hand makes the unfolded protein more susceptible to trypsin’s hydrolytic action. The effect of higher temperatures in the formation of whey protein aggregates has been reported before [31]. Havea, Singh. and Creamer [31] mentioned that at higher temperatures, whey proteins unfold and interact to form aggregates; this phenomenon is higher in cheese whey than in acid whey, probably due to the minerals present. 

According to the ANOVA, the best trypsin hydrolytic conditions to obtain higher antioxidant capacity and ACEI inhibition were 52 °C and pH 8.2. The pH value agrees with the reported optimal range for this enzyme. The temperature was higher than the optimal reported (37 °C). Chelulei Cheison et al. [32] reported that trypsin could hydrolyze β-lactoglobulin outside its optimum temperature and alkaline pH, producing peptides with predictable biofunctionalities.

### 4.1. Antioxidant Capacity

Whey is a byproduct in the cheese production process. Since whey has a high organic content, it is an environmental problem of the dairy industry. In this study, it was possible to obtain bioactive peptides showing a maximum DPPH-scavenging activity of 26% from the whey discarded from panela (white) cheese production. This value is similar to the hydrophilic peptide fractions obtained by Şanlıdere Aloğlu and Öner [33] from yogurt. On the other hand, lower antioxidant activity was reported for whey protein hydrolysates in low molecular weight fractions, corresponding to water soluble-peptides [34]. Trypsin whey fractions obtained by Önay-Uçar et al. [35] showed not only free radical scavenging capacity, but also high capacity to chelate Cu. These activities were attributed to low molecular weight peptides obtained during Sephadex fractionation. 

In recent years, special attention has been focused on the production of whey peptides with radical scavenging capacity, which can be applied not only as food ingredients, but also as nutraceuticals [5,14,36]. Moreover, several enzymes have been used to obtain bioactive peptides from whey, including digestive [16,35], microbial [37], commercial mixtures [14,38,39], and even direct microbial fermentation [36].

The hydrophilic and AA fractions were highly correlated with antioxidant activity (Table 2). Moreover, around half of the peaks in the hydrophilic fraction were positively correlated with antioxidant activity. As observed by several authors, low molecular weight peptides, which were water soluble, showed high antioxidant capacity, measured by different in vitro methods such as ORAC (Oxygen Radical Absorbance Capacity), ABTS (2,2′-Azino-bis(3-ethylbenzothiazoline-6-sulfonic acid)), and DPPH [14,33,35,37,38,39]. 

Our results showed that peptides present in the hydrophobic fraction also have antioxidant capacity. However, only nine peaks were positively correlated with DPPH scavenging capacity. DPPH is a free radical soluble in organic solvents such as methanol; several authors suggest that due to it scarce solubility in water, the antioxidant capacity of hydrophilic antioxidants such as water-soluble peptides, could be underestimated [33]. However, in this study, a range of 4.76–26.20% discoloration was obtained. The variation in antioxidant capacity was strongly modified by hydrolysis conditions (pH and temperature), suggesting that at least in this case, the DPPH solution was mixed with peptides, either hydrophilic or hydrophobic, and dissolved in water media without any problem. 

Lacobacterial whey fermentation produced high molecular weight peptides, which showed antioxidant capacity [36], expressed as Trolox µM equivalent. Obtention of peptides with a high molecular weight in the hydrophobic fraction is not a barrier to get antioxidant capacity. 

### 4.2. ACEI Activity

The average ACEI activity observed for the peptides was 0.79 times the average activity of captopril (Table 1). The peptides’ activities were higher than captopril activity since the mean hydrolysates concentration was only 0.28 mg/mL compared with 5 mg/mL captopril. Captopril is a highly specific ACE inhibitor that acts in the enzyme’s active center [40].

The ACEI activity reached in our study was higher than the activities reported before [7,10]. Abubakar, Saito, Kitazawa, Kawai, and Itoh [10] reported 56.7% ACEI activity in dry commercial cheese whey hydrolysates obtained at the best conditions for trypsin hydrolysis (pH 8, 37 °C). As shown in Figure 1d, under the reported best trypsin hydrolytic conditions, ACEI activity was lower than at 50%. Espejo-Carpio, De Gobba, Guadix, Guadix, and Otte [7] reported 33% ACEI activity after hydrolysis of caprine milk filtrate proteins with trypsin. These authors carried out the hydrolysis under the same conditions of pH and temperature, whereas we observed the highest ACEI activity after only 4 h; this could explain the observed differences in ACEI activity. 

## 5. Conclusions

The cheese whey discarded after Mexican white cheese production was successfully used as a raw material to produce bioactive peptides. Trypsin hydrolysis of concentrated cheese whey at 52 °C and pH 8.2 made it possible to obtain peptides with higher antioxidant capacity and ACEI inhibitory activity. A highly significant correlation was found between antioxidant capacity and ACEI activity. Highly significant correlations were found for both biological activities with different peptides, mostly in the AA and HI fractions, but significant correlations were also found for the HO fraction. Additional studies are necessary to fractionate the obtained peptides to evaluate individual activities and to identify specific peptides. Further research is also needed to evaluate the suitability of cheese whey from other kinds of cheese production to produce bioactive peptides.

## Figures and Tables

**Table 1:** Analysis of variance results with an α = 0.05.

Parameter	Factor (*p*-Value) ^a^		Mean	Minimum	Maximum
	pH	Temperature			
% Hydrolysis	0.002 **	0.230	66.67	32.82	100.34
Soluble Protein ^b^	0.010 **	0.000 ***	0.28	0.05	0.46
Antioxidant capacity ^c^	0.035 *	0.000 ***	12.04	4.76	26.20
Δ ACE inhibition ^d^	0.000 ***	0.246	0.79	0.68	0.89
AA fraction	0.438	0.171	25,412.87	13,200.70	35,041.30
Hydrophilic fraction (HI)	0.161	0.000 ***	15,602.86	7364.70	27,454.90
Hydrophobic fraction (HO)	0.890	0.563	23,738.38	409.50	372,127.00
Ratio HI:HO	0.139	0.071	2.94	0.03	20.12

^a^ Significant difference at * *p* < 0.05, ** *p* < 0.01, and *** *p* < 0.001. ^b^ Expressed as mg/mL. ^c^ Expressed as the 2,2- diphenyl-1-picrylhydrazyl (DPPH) % discoloration. ^d^ Ratio between Angiotensin converting enzyme (ACE) peptides inhibition/captopril inhibition.

**Table 2:** Correlation table of biological activity of the produced peptides with an α = 0.05.

Variable ^a^	RT	Antioxidant Capacity ^b^	Δ ACE Inhibition ^c^	Variable	RT	Antioxidant Capacity ^b^	Δ ACE Inhibition ^c^
Δ ACE inhibition ^c^		0.516 *		P61	22.603	0.667 **	0.518 *
AA fraction		0.333	0.204	P62	22.899	−0.290	−0.547 *
Hydrophilic fraction (HI)		0.770 ***	0.706 **	P73	26.790	0.541 *	0.512 *
Hydrophobic fraction (HO)		−0.231	0.005	P74	27.345	0.745 ***	0.374
Ratio HI:HO		−0.069	−0.516 *	P75	27.623	−0.526 *	−0.008
P2	2.045	0.379	0.513 *	P76	28.114	0.631 **	0.501 *
P3	2.432	−0.789 ***	−0.257	P78	28.704	0.563 *	0.121
P8	3.759	0.346	0.576 *	P80	29.626	0.498 *	0.363
P10	4.103	0.720 ***	0.484 *	P81	29.734	0.546 *	0.243
P11	4.857	0.746 ***	0.573 *	P82	30.868	0.636 **	0.575 *
P12	5.689	0.446	0.601 **	P86	32.838	0.641 **	0.464
P13	6.133	0.535 *	0.565 *	P87	33.339	0.647 **	0.533 *
P18	8.316	0.524 *	0.496 *	P89	34.293	0.471 *	0.711 ***
P19	8.943	0.591 **	0.616 **	P90	34.590	−0.271	0.022
P22	10.204	0.548 *	0.324	P91	34.848	0.390	0.590 **
P23	10.547	0.720 ***	0.498 *	P92	35.479	0.611 **	0.633 **
P27	12.367	0.359	0.475 *	P98	38.279	0.580 *	−0.014
P28	13.195	0.389	0.514 *	P99	38.950	0.481 *	0.018
P31	14.097	0.392	0.569 *	P100	39.193	0.035	0.476 *
P34	14.893	0.612 **	0.051	P102	39.876	0.590 *	0.348
P35	15.050	0.048	0.537 *	P106	41.778	0.241	0.494 *
P37	15.698	0.568 *	0.426	P107	42.052	0.605 **	0.126
P39	16.190	0.519 *	0.216	P109	43.430	0.729 ***	0.563 **
P45	17.733	0.796 ***	0.561 *	P110	44.162	0.771 ***	0.168
P46	18.131	0.555 *	0.450	P111	44.881	0.692 **	0.250
P48	18.768	0.520 *	0.593 **	P114	46.743	0.831 ***	0.590 **
P51	19.534	0.580 *	0.595 **	P116	48.529	0.290	0.503 *
P54	20.964	0.466	0.687 **	P119	53.330	0.344	0.646 **
P56	21.355	0.422	0.475 *	P120	58.930	0.335	0.506 *

^a^ Significant correlation at * *p* < 0.05, ** *p* < 0.01, and *** *p* < 0.001. ^b^ Expressed as DPPH% discoloration. ^c^ Ratio between ACE peptides inhibition/captopril inhibition.
